# Renal haemodynamic, microcirculatory, metabolic and histopathological responses to peritonitis-induced septic shock in pigs

**DOI:** 10.1186/cc7164

**Published:** 2008-12-24

**Authors:** Jiri Chvojka, Roman Sykora, Ales Krouzecky, Jaroslav Radej, Veronika Varnerova, Thomas Karvunidis, Ondrej Hes, Ivan Novak, Peter Radermacher, Martin Matejovic

**Affiliations:** 1 Intensive care unit, 1^st ^Medical Department, Charles University Medical School and Teaching Hospital Plzen, alej Svobody 80, Plzen, 304 60, Czech Republic; 2 Department of Pathology, Charles University Medical School and Teaching Hospital Plzen, Czech Republic, alej Svobody 80, Plzen, 304 60, Czech Republic; 3 Sektion Anästhesiologische Pathophysiologie und Verfahrensentwicklung, Universitätsklinikum, Parkstraße 11, Ulm, 890 73, Germany

## Abstract

**Introduction:**

Our understanding of septic acute kidney injury (AKI) remains incomplete. A fundamental step is the use of animal models designed to meet the criteria of human sepsis. Therefore, we dynamically assessed renal haemodynamic, microvascular and metabolic responses to, and ultrastructural sequelae of, sepsis in a porcine model of faecal peritonitis-induced progressive hyperdynamic sepsis.

**Methods:**

In eight anaesthetised and mechanically ventilated pigs, faecal peritonitis was induced by inoculating autologous faeces. Six sham-operated animals served as time-matched controls. Noradrenaline was administered to maintain mean arterial pressure (MAP) greater than or equal to 65 mmHg. Before and at 12, 18 and 22 hours of peritonitis systemic haemodynamics, total renal (ultrasound Doppler) and cortex microvascular (laser Doppler) blood flow, oxygen transport and renal venous pressure, acid base balance and lactate/pyruvate ratios were measured. Postmortem histological analysis of kidney tissue was performed.

**Results:**

All septic pigs developed hyperdynamic shock with AKI as evidenced by a 30% increase in plasma creatinine levels. Kidney blood flow remained well-preserved and renal vascular resistance did not change either. Renal perfusion pressure significantly decreased in the AKI group as a result of gradually increased renal venous pressure. In parallel with a significant decrease in renal cortex microvascular perfusion, progressive renal venous acidosis and an increase in lactate/pyruvate ratio developed, while renal oxygen consumption remained unchanged. Renal histology revealed only subtle changes without signs of acute tubular necrosis.

**Conclusion:**

The results of this experimental study argue against the concept of renal vasoconstriction and tubular necrosis as physiological and morphological substrates of early septic AKI. Renal venous congestion might be a hidden and clinically unrecognised contributor to the development of kidney dysfunction.

## Introduction

Despite the fact that acute kidney injury (AKI) in critically ill patients is predominantly caused by sepsis and septic shock [1], the pathophysiology of septic AKI is still poorly understood [2]. Although renal vasoconstriction and consequent renal ischaemia and acute tubular necrosis (ATN) occupy a central role in AKI development in hypodynamic states, during sepsis the role and character of haemodynamic alterations within the kidney still remain controversial [2-4].

It must be stressed that the majority of studies reporting a reduction in renal blood flow were derived from fairly heterogenous, short-term and mostly hypodynamic models characterised by a reduced cardiac output, which therefore only have a limited resemblance with human pathophysiology [2,3]. By contrast, utilising clinically more relevant models of hyperdynamic sepsis in sheep, Langenberg and colleagues have recently challenged the conventional presumption of renal vasoconstriction as a prerequisite for the development of AKI during hyperdynamic bacteraemia in a sheep model [5-8]. As suggested earlier [9], the authors provided 'proof of concept' that septic AKI may represent a unique form of hyperaemic AKI [2]. However, further research is needed to establish whether this concept is valid in other clinically relevant models of sepsis-induced AKI. [8,10].

In addition, the vast amount of experimental studies report on haemodynamic changes only, without providing their relationship to microcirculatory, metabolic and histopathological responses. Prompted by these facts, we dynamically assessed the pattern of renal haemodynamics in a long-term porcine model of progressive hyperdynamic sepsis induced by faecal peritonitis. Furthermore, a potential link between renal haemodynamics and renal cortex microcirculatory, metabolic and histological changes was simultaneously analysed.

## Materials and methods

Animal handling was in accordance with the European Directive for the Protection of Vertebrate Animals Used for Experimental and Other Scientific Purposes (86/609/EU). The experiments were approved by the Committee for Experiments on Animals of the Charles University Medical School, Plzen, Czech Republic.

### Animals and preparations

Fourteen domestic pigs with a median body weight of 32 kg (range: 27 to 35 kg) were investigated. Anaesthesia was induced with intravenous atropine (0.5 mg), propofol 2% (1 to 2 mg/kg) and ketamine (2.0 mg/kg). Animals were mechanically ventilated (fraction of inspired oxygen 0.4; positive end-expiratory pressure 5 to 10 cm H_2_O; tidal volume 10 ml/kg; respiratory rate was adjusted to maintain arterial partial pressure of carbon dioxide (PCO_2_) between 4.0 to 5.0 kPa). Surgical anaesthesia was maintained with continuous intravenous thiopental (10 mg/kg/hour) and fentanyl (10 to 15 μg/kg/hour). Thereafter, continuous thiopental (5 mg/kg/hour) and fentanyl infusions (5 μg/kg/hour) were maintained until the end of the experiment (in total 30 hours of anaesthesia: 8 hours surgery and stabilisation period, 22 hours experiment). Muscle paralysis was achieved with pancuronium (4 to 6 mg/hour). Infusion of Plasma Lyte solution (Baxter Healthcare, Deerfield, IL, United States) 15 ml/kg/hour was administered during surgery and than reduced to 7 ml/kg/hour as a maintenance fluid. To maintain arterial blood glucose levels between 4.5 and 7 mmol/l during the whole experiment, 20% glucose was infused.

Central venous and pulmonary artery catheters for monitoring of systemic haemodynamics, blood sampling and drug infusions were placed via jugular veins. One femoral arterial catheter was placed for blood pressure recording and blood sampling, and a fibre-optic one for thermal-dye double-indicator dilution measurements (only in septic animals). After performing midline laparotomy, a precalibrated ultrasound flow probe (Transonic Systems, Ithaca, NY) was placed around the left renal artery. Renal cortex microcirculation was monitored by placing Laser Doppler probe (PF 404, Suturable angled probe, Perimed, Jarfalla, Sweden) directly over the renal cortex. A double-lumen catheter was inserted into the left renal vein for renal venous pressure measurements and blood sampling. Two drains were used for peritonitis induction and ascites drainage. Then, the abdominal wall was closed and epicystostomy under ultrasound control was performed percutaneously for urine collection. The pigs were allowed to stabilise after the surgery for a period of six hours before baseline data collection and measurements were performed.

### Haemodynamic measurements and calculations

The measurement of systemic haemodynamics included cardiac output (CO), systemic vascular resistance (SVR), intrathoracic blood volume (ITBV) and filling pressures of both ventricles (central venous pressure (CVP), pulmonary artery occlusion pressure (PAOP)). Arterial, mixed venous and renal blood samples were analysed for pH, partial pressure of oxygen (pO_2_), pCO_2 _and for haemoglobin oxygen saturation. Systemic oxygen delivery, systemic oxygen uptake and renal oxygen delivery and oxygen uptake were derived from the appropriate blood gases and flow measurements. Renal vascular resistance (RVR) was calculated according to the formula:

RVR = mean arterial pressure (MAP; mmHg) – renal venous pressure (mmHg)/renal blood flow (l.min^-1^)

### Blood and tissue samples

Arterial and renal venous lactate (L) and pyruvate (P) concentrations were measured. Arterial blood samples were analysed for plasma creatinine, tumour necrosis factor-α (TNF-α; immunoassay) and interleukin-6 (IL-6; immunoassay) levels [11,12]. Oxidative and nitrosative stress were evaluated by measuring concentrations of arterial thiobarbituric acid reactive species (TBARS; spectrophotometry) and arterial nitrate/nitrite (NOx; colorimetric assay) [11,12]. To correct for dilutional effects resulting from volume resuscitation, the levels of NOx, TBARS, IL-6 and TNF-α were normalised with plasma protein content [11,12]. At the end of the experiment, the left kidney was harvested for H&E staining and semiquantitative analysis of the kidney tissue damage was performed in a blinded fashion by a certified nephropathologist.

### Protocol

Following a recovery period of six hours, baseline measurements were recorded and pigs were randomised to sham operated (control, n = 6) or to septic group (AKI, n = 8). In the septic group, faecal peritonitis was induced by inoculating 0.5 g/kg of autologous faeces suspended in 200 ml saline through the drains into the abdomen. After 12, 18 and 22 hours after the induction of peritonitis the next set of measurements and data collection were performed. In addition to an infusion of the Plasma Lyte solution, 6% hydroxyethylstarch 130 kD/0.4 (Voluven 6%, Fresenius Kabi Deutschland GmbH, Bad Homburg, Germany) was infused at a rate of 10 ml/kg/hour (7 ml/kg/hour if CVP or PAOP ≥ 18 mmHg) to maintain cardiac filling pressures at 12 mmHg or above. Continuous intravenous noradrenaline was administered if MAP fell below 70 mmHg and titrated to maintain MAP above 65 mmHg. When the last set of data had been obtained, the animals were euthanased by potassium chloride injection under deep anaesthesia and section was performed.

### Statistical analysis

All values shown are median and interquartile ranges. After exclusion of normality using Kolmogorov-Smirnov test, differences within each group before and after induction of peritonitis were tested using a Friedman analysis of variance on ranks and, subsequently, a Dunn's test for multiple comparisons with Bonferroni's correction. The Mann-Whitney rank sum test was performed to compare data between treatment groups. A p < 0.05 was regarded as statistically significant.

## Results

There were no statistically significant differences in any measured variables between the sham-operated and peritonitis-induced pigs at baseline.

### Systemic variables

Haemodynamic and oxygen exchange parameters, inflammatory responses, oxidative and nitrosative stress, and other laboratory parameters are summarised in Table 1. Faecal peritonitis induced a hyperdynamic circulatory state with an increased cardiac output and low SVR. All pigs in the peritonitis group needed noradrenaline (median dose of 1.8 μg/kg/min) to maintain MAP above 65 mmHg. The median time to development of arterial hypotension was 16 hours. Adequate fluid resuscitation was ensured by monitoring cardiac filling pressures that were significantly increased over time in septic animals, while intrathoracic blood volume was well maintained (baseline 23 (22 to 24), 22 hours of sepsis 24 (19 to 34) ml/kg). The increased CO resulted in a significant rise of systemic oxygen delivery, while systemic oxygen consumption remained unchanged. The peritonitis-induced sepsis caused a significant fall of arterial pH and markedly increased plasma levels of TNF-α and IL-6. Overproduction of NOx in this model was documented by a significant increase in arterial NOx levels. These changes were accompanied by a remarkable increase of TBARS levels providing the evidence for oxidative stress.

**Table 1:** Systemic variables

	Group	Baseline	12 hours	18 hours	22 hours
MAP	Control	91 (86 to 95)	82.5 (71 to 88)	77 (70 to 81)	77 (64 to 85)
(mmHg)	AKI	94 (89 to 97)	80 (72.5 to 93)	75 (68 to 83)*	72 (66 to 76)*

MPAP	Control	25 (20 to 26)	26 (23 to 27)	25 (24 to 26)	26 (25 to 29)
(mmHg)	AKI	24 (21 to 28)	31 (28 to 37)*§	42 (34 to 46)*§	44 (41 to 47)*§

CO	Control	92 (83 to 110)	98 (88 to 109)	87 (79 to 100)	88 (79 to 102)
(ml/kg)	AKI	79 (64 to 102)	113 (95 to 164)§	140 (116 to 178)*§	174 (120 to 191)*§

SVR	Control	2697 (1508 to 2914)	2007 (1913 to 2093)	1930 (1876 to 1965)	1950 (1670 to 2289)
(dyne.s.cm-5)	AKI	2595 (1972 to 2809)	1523 (968 to 1917)*§	964 (683 to 1263)*§	757 (600 to 852)*§

CVP	Control	8 (8 to 9)	11 (10 to 13)*	13 (12 to 15)*	13 (12 to 15)*
(mmHg)	AKI	10 (9 to 13)	13 (12 to 15)*	16 (14 to 18)*	18 (16 to 19)*§

PAOP	Control	8 (7 to 10)	11 (8 to 12)	12 (10 to 13)	12 (10 to 17)
(mmHg)	AKI	10 (9 to 12)	12 (11 to 15)*	16 (14 to 17)*§	16 (16 to 17)*

DO2	Control	11 (11 to 12)	11 (9 to 12)	10 (9 to 12)	10 (9 to 12)
(ml/min/kg)	AKI	11 (9 to 13)	16 (14 to 27)§	16 (14 to 19)§	19 (11 to 25)

VO2	Control	5 (4 to 6)	6 (5 to 6)	5 (5 to 6)	5 (5 to 5)
(ml/min/kg)	AKI	5 (5 to 6)	6 (5 to 8)	6 (5 to 6)	6 (5 to 8)

pH	Control	7.58 (7.55 to 7.59)	7.56 (7.52 to 7.58)	7.57 (7.54 to 7.60)	7.58 (7.50 to 7.61)
	AKI	7.54 (7.52 to 7.60)	7.45 (7.43 to 7.49)*§	7.41 (7.20 to 7.47)*§	7.31 (7.08 to 7.36)*§

IL-6	Control	2 (1 to 3)	1 (0 to 1)	1 (0 to 1)	1 (0 to 2)
(pg/ml/g protein)	AKI	4 (1 to 8)	41 (29 to 201)*§	240 (111 to 602)*§	384 (163 to 1405)*§

TNF-α	Control	0 (0 to 0)	1 (1 to 2)	2 (1 to 2)	1 (1 to 2)
(pg/ml/g protein)	AKI	1 (1 to 2)	10 (5 to 16)*§	17 (8 to 28)*§	21 (8 to 33)*§

TBARS	Control	17 (16 to 19)	19 (17 to 28)	22 (19 to 30)	23 (16 to 25)
(nmol/g protein)	AKI	18 (15 to 24)	63 (44 to 93)*§	90 (71 to 117)*§	78 (68 to 108)*§

Plasma nitrate+nitrite levels	Control	1 (1 to 1)	1 (0 to 1)*	1 (0 to 1)*	1 (1 to 1)
(μmol/g protein)	AKI	1 (1 to 1)	1 (1 to 2)*§	2 (1 to 2)*§	1 (1 to 2)*

CO = cardiac output, CVP = central venous pressure, DO2 = oxygen delivery, IL = interleukin, MAP = mean arterial pressure, MPAP = mean pulmonary artery pressure, PAOP = pulmonary artery occlusion pressure, SVR = systemic vascular resistance, TBARS = thiobarbituric acid reactive species, TNF = tumour necrosis factor, VO2 = oxygen uptake.

Control = sham operated group; AKI = peritonitis induced group

* significant difference within each group versus baseline (p < 0.05); §significant difference between groups (p < 0.05). Data are median and 25th and 75th quartiles.

### Renal haemodynamics, microcirculation, metabolism, function and histology

**Figure 1:**
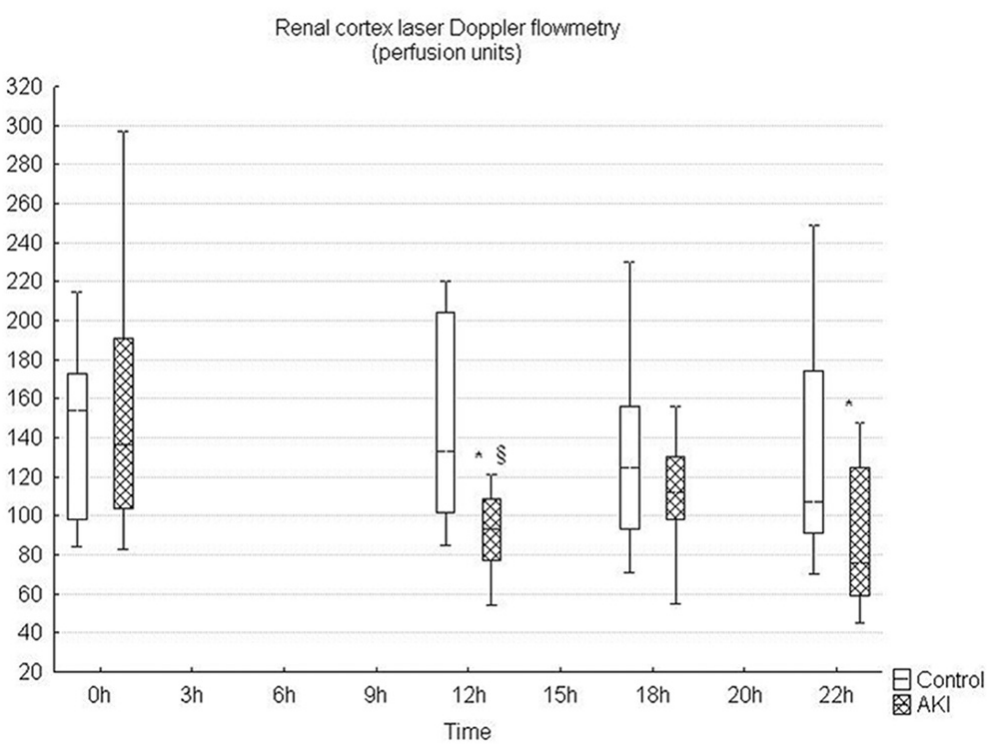
**Renal cortex microcirculation during the time course of sepsis showing early deterioration of microcirculation perfusion in the peritonitis group (AKI)**. * significant difference within each group versus baseline (p < 0.05); §significant difference between groups (p < 0.05).

**Figure 2:**
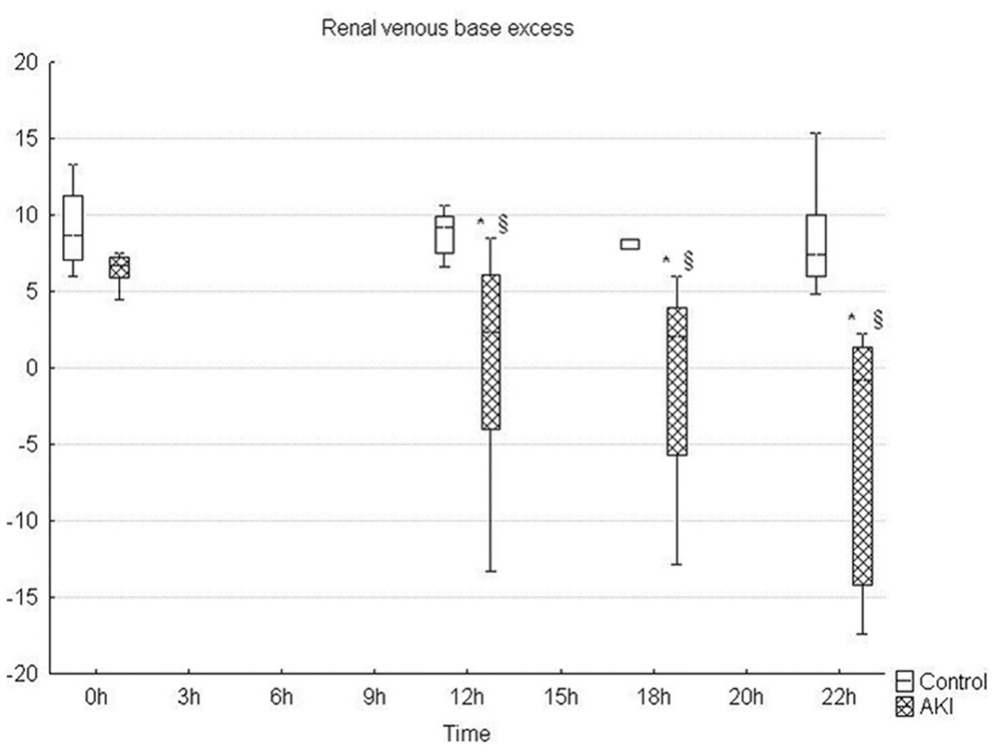
**Progressive renal venous acidosis during the time course of sepsis**. * significant difference within each group versus baseline (p < 0.05); §significant difference between groups (p < 0.05). AKI = peritonitis induced group.

**Figure 3:**
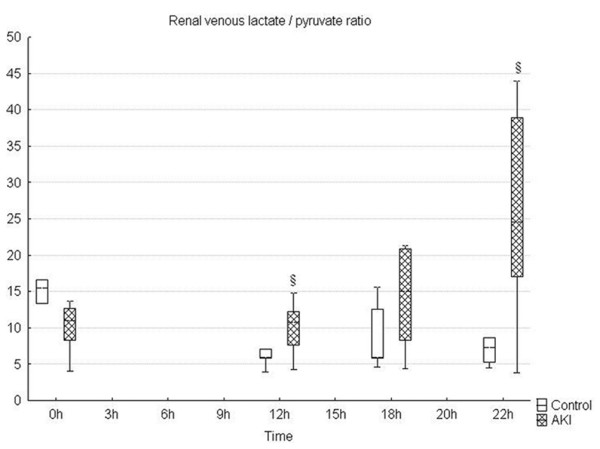
**Gradual worsening of lactate/pyruvate ratio during the time course of sepsis**. * significant difference within each group versus baseline (p < 0.05); §significant difference between groups (p < 0.05). AKI = peritonitis induced group.

**Figure 4:**
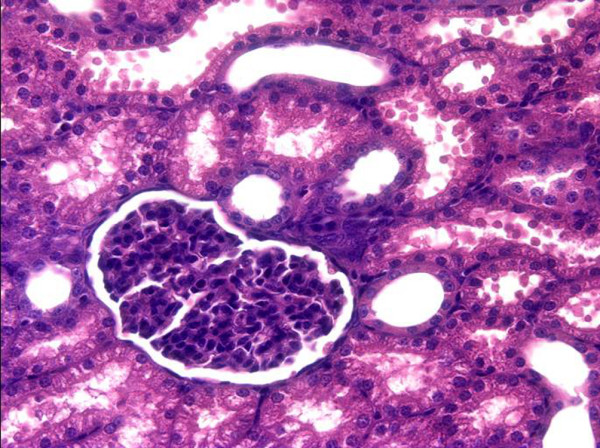
**Representative histological image of a control kidney**.

**Figure 5:**
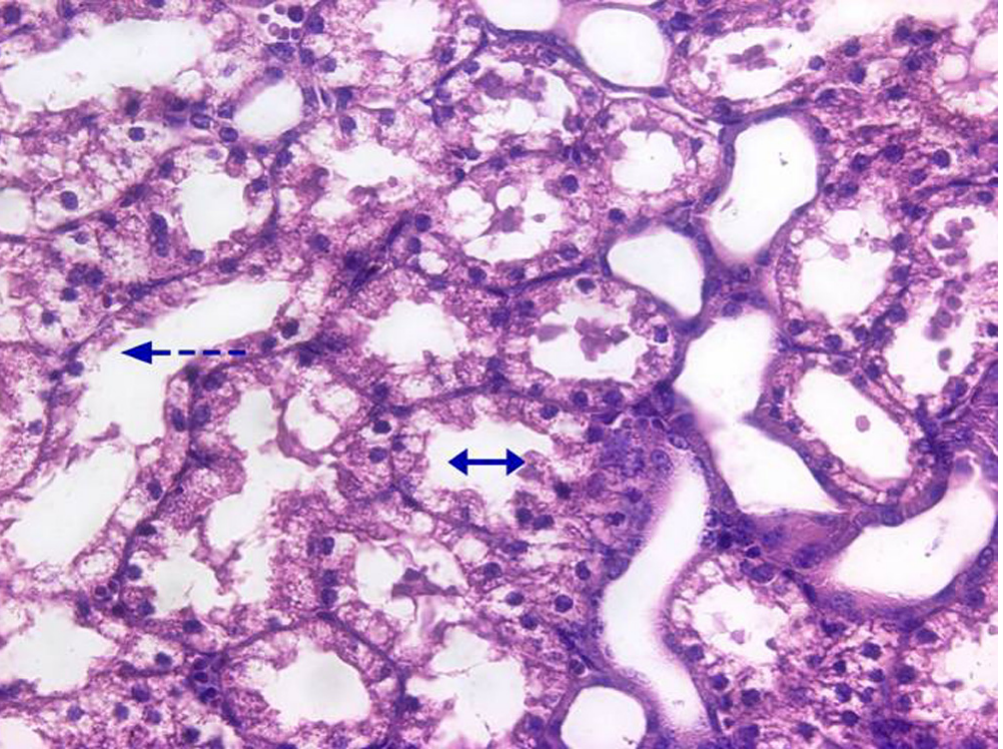
**Representative histological image of a septic kidney**. Arrows showing epithelial cells vacuolisation with damage of brush border.

The parameters of renal haemodynamics, oxygen exchange, cortex microcirculation, metabolic and acid-base status, as well as kidney function and histomorphology are presented in Table 2 and in Figures 1 to 5. Renal blood flow remained unchanged during hyperdynamic sepsis, with only minor decline at the end of the experiment compared with baseline values. Nevertheless, there were no intergroup differences throughout the whole experiment. RVR did not change either and even decreased at 18 hours of sepsis compared with the control group. Although renal artery pressure was maintained with noradrenaline, renal perfusion pressure significantly decreased in the AKI group as a result of gradually increased renal venous pressure. Despite maintained renal blood flow, renal cortex microcirculation decreased early and this deterioration persisted until the end of the experiment (Figure 1).

**Table 2:** Renal haemodynamics, oxygen exchange and acid base balance

	Group	Baseline	12 hours	18 hours	22 hours
Delta creatinine	Control	0 (0 to 0)	-1.5 (-7 to 0)	0.5 (-4 to 11)	2 (-1 to 11)
(μmol/l)	AKI	0 (0 to 0)	8 (-12 to 27)	23 (-3 to 46)	31 (18 to 60)*§

RBF	Control	5 (5 to 9)	4 (4 to 6)	4 (3 to 6)	4 (3 to 6)
(ml/kg)	AKI	6 (4 to 8)	5 (4 to 8)	7 (5 to 7)	4 (3 to 4)*

RVP	Control	9 (8 to 10)	15 (12 to 16)	13 (12 to 14)	12 (11 to 20)
(mmHg)	AKI	13 (11 to 15)	14 (12 to 16)	16 (15 to 19)*§	21 (18 to 25)*

RPP	Control	82 (77 to 89)	68 (59 to 72)	65 (58 to 70)	70 (44 to 75)
(mmHg)	AKI	84 (76 to 87)	71 (59 to 78)*	63 (55 to 65)*	51 (48 to 58)*

Renal DO2	Control	0.63 (0.55 to 0.93)	0.48 (0.42 to 0.68)	0.47 (0.39 to 0.70)	0.40 (0.34 to 0.63)
(ml/min/kg)	AKI	0.81 (0.59 to 0.97)	0.87 (0.46 to 1.16)	0.8 (0.53 to 0.87)	0.50 (0.22 to 0.58)

Renal VO2	Control	0.17 (0.13 to 0.22)	0.14 (0.13 to 0,24)	0.18 (0.13 to 0.21)	0.19 (0.14 to 0.22)
(ml/min/kg)	AKI	0.21 (0.14 to 0.24)	0.17 (0.08 to 0.23)	0.23 (0.21 to 0.23)	0.17 (0.12 to 0.24)

Renal ER	Control	28 (22 to 33)	33 (31 to 39)	33 (31 to 39)	32 (30 to 52)
(%)	AKI	26 (22 to 27)	26 (16 to 32)	25 (21 to 45)	37 (34 to 58)*

Renal venous pH	Control	7.54 (7.53 to 7.55)	7.5 (7.48 to 7.53)	7.53 (7.51 to 7.56)	7.50 (7.47 to 7.57)
	AKI	7.53 (7.5 to 7.57)	7.42 (7.42 to 7.47)*§	7.4 (7.21 to 7.47)*§	7.31 (7.01 to 7.35) §

UO	Control	4 (3 to 6)	3 (2 to 6)	4 (3 to 6)	4 (3 to 5)
(ml/kg/h)	AKI	2 (2 to 3)§	2 (1 to 4)	2 (1 to 2)§	1 (1 to 2)*§

DO2 = oxygen delivery, ER = extraction ratio, RBF = renal blood flow, RPP = renal perfusion pressure, RVP = renal venous pressure, UO = urine output, VO2 = oxygen uptake.

Control = sham operated group; AKI = peritonitis induced group

* significant difference within each group vs. baseline (p < 0.05); §significant diference between groups (p < 0.05) Data are median and 25th and 75th quartiles.

Microvascular alterations were associated with a marked metabolic stress of the kidney as documented by a significant development of renal venous metabolic acidosis and progressively increased L/P ratio (Figures 2 and 3). The renal oxygen extraction increased at the end of the experiment in septic animals, without changes in renal oxygen consumption. Progressive sepsis caused renal dysfunction as evidenced by significant changes in serum creatinine levels (Table 2). In addition, urine output significantly decreased in the AKI group over time. Only minor histological changes encompassing mild brush-border loss and vacuolisation of tubular cells were present at 22 hours of the experiment on kidney histology. No signs of ATN or tubular cast formation were found. Representative images of control and septic kidney are shown in Figures 4 and 5.

## Discussion

In this clinically relevant model of hyperdynamic septic shock AKI developed without apparent renal vasoconstriction, renal oxygen consumption did not change and renal histology revealed only subtle changes despite significant kidney cortex microvascular and metabolic stress. Renal venous congestion might contribute to the pathogenesis of septic AKI.

The renal haemodynamic, microvascular and metabolic responses to and morphological sequelae of sepsis remain inconsistent because of marked heterogeneity attributable to the use of different species, models of sepsis, experimental settings and supportive treatment. Hence, the next fundamental step to understand the pathophysiology of septic AKI is the use of animal models designed to meet the criteria of human sepsis/septic shock [13,14]. However, the majority of studies have been derived from very heterogenous, short-term and mostly hypodynamic models characterised by a reduced CO. By contrast, the sepsis model used in our study replicates many of the features of adequately resuscitated human septic shock (i.e. hyperdynamic circulation, inflammatory response accompanying with nitrosative, oxidative and metabolic stress). The substantial instrumentalisation used offers a broad insight into organ haemodynamic and metabolic pathways, thereby making it an appealing sepsis model in studies of AKI. Moreover, this is underpinned by the fact that the pig kidney is more similar to the human kidney than that of dog, rat or mice because of similar renal anatomy, architecture and lymphatic pattern, urinary concentrating ability, tolerance to ischaemia and medullary thickness [15,16].

To our knowledge, our study is the first to tackle the issue of directly measured renal venous pressure allowing both the determination of RVR and true renal perfusion pressure in a large animal sepsis model. In keeping with recent reports [5,6,8], our study provides further evidence against the widely held concept that sepsis increases RVR [1]. Importantly, it is apparent from the present study that, at least in this model, renal venous congestion leading to decreased renal perfusion pressure might play an important role in mediating fall in glomerular filtration despite clinically acceptable MAP and CO.

It is amazing that there are no animal studies that provide information on the behaviour of renal venous pressure in their sepsis models. The combined impact of both venous congestion due to elevated right atrial pressure and sepsis-induced capillary leak promoting the development of tissue oedema and abdominal hypertension could explain the elevated renal venous pressure and associated reduction in the filtration gradient. Of note, our data suggests that the assumption that renal perfusion pressure is essentially equal to MAP might not be valid under conditions of severe capillary leak and aggressive fluid resuscitation.

The well-preserved renal blood flow does not guarantee adequate perfusion to microvascular beds. Indeed, peritonitis-induced sepsis caused significant reduction in cortical microvascular perfusion in our model, supporting the emerging evidence that renal microvascular dysfunction may be a culprit of septic AKI [17]. Within the limitations arising from renal cortex laser Doppler flowmetry measurements, we can unambiguously determine neither the most affected part of the nephron nor the fate of intrarenal distribution of blood flow in deeper cortex layers and medulla in our model. Nevertheless, recent long-term rodent models of sepsis-induced acute renal failure demonstrated marked decline in cortical peritubular capillary perfusion [18-20] that was associated with tubular redox stress and preceded the development of renal failure [19]. In addition, the distribution of blood flow from the cortex towards medulla has been suggested by several studies [21-23], although contradictory results have also been reported [24].

Only a few studies with conflicting data have been performed investigating simultaneous renal haemodynamics and oxygen [23,25-28]. In our study, the apparent kidney metabolic stress as evidenced by gradually worsened renal venous L/P ratio (a marker of redox state) and acid base status occurred despite unchanged renal oxygen consumption. The design of the present study does not allow conclusions to be drawn about what processes are responsible for altered kidney energy metabolism. Nevertheless, taking into account an increased renal oxygen extraction at the end of the experiment, our results could indicate that these metabolic alterations may be attributable to the deterioration in microcirculatory perfusion and related tissue hypoxia. Of note, highly heterogenous renal tissue perfusion and oxygen consumption within the kidney make any extrapolation of the total oxygen uptake measurement potentially erroneous and regions or energy requiring pathways (e.g. tubular sodium reabsorption) suffering from hypoxia might have been overlooked [29]. In support of this notion, in endotoxaemic rats, Johannes and colleagues recently provided the evidence for the presence of microvascular hypoxic areas, even though renal oxygen consumption was not significantly reduced and no hypoxia was detected in the average microcirculatory pO2 measurements [30]. Finally, Porta and colleagues recently showed that kidney mitochondrial function was preserved in a prolonged porcine endotoxaemia with well-maintained renal blood flow [28], making the disturbed cellular energy machinery independent of tissue oxygen availability a less plausible explanation for the renal metabolic stress.

Analogous to the renal hypoperfusion paradigm in septic AKI, ATN is generally regarded as the most frequent mechanism of renal failure in critically ill patients [1]. However, there is no published study in septic patients, that would provide conclusive histopathological evidence for the presence of ATN in sepsis-induced AKI and very few experimental studies simultaneously evaluate physiological features of AKI and underlying histopathological changes allowing data to be put into the relevant complex picture. In our model, only subtle histological changes without any signs of ATN occurred despite marked microvascular and metabolic changes. Admittedly, a longer duration of the experiment could have been required for the development of more severe kidney dysfunction and corresponding histological changes. On the other hand, our results are consistent with a recently published systematic review showing only mild, non-specific changes in the majority of clinical and experimental studies [31].

Our study has several limitations. Despite a gradual severity of the septic process, the pigs developed relatively mild AKI, probably as a result of early and aggressive haemodynamic management. Nevertheless, even small changes in serum creatinine (delta 30 μmol/l in our study) achieved within 22 hours suggest significant renal injury, confirmed by some histological evidence. One could also argue that fluid resuscitation with a large dose of hydroxyethylstarch could contribute to the renal dysfunction in this model. However, the available data still remains inconclusive and the safety profile of a new colloid generation as used in our study (6% hydroxyethylstarch 130/0.4) needs to be further clarified. Due to logistical limitations, other important variables of renal function, such as creatinine clearance or tubular reabsorption functions, were not measured. In addition, we did not directly measure intra-abdominal pressure and the absence of techniques to precisely assessing intraglomerular and peritubular microvasculature and tissue oxygenation/energetics do not allow any robust conclusions to be drawn. Finally, the long-term effects of sepsis over several days might give different results.

## Conclusion

Within the boundaries of the limitations, the results of our study support the recent evidence arguing against the concept of renal vasoconstriction and ATN as physiological and morphological substrates of early septic AKI and show that renal venous congestion might be a hidden and clinically unrecognised factor in the development of kidney dysfunction.

## Key messages

• The results of this experimental study argue against the concept of renal vasoconstriction in early sepsis-induced kidney dysfunction.

• Despite maintained renal perfusion significant renal cortex microvascular and metabolic stress developed very early in the course of AKI.

• Kidney oxygen extraction capabilities remained well-maintained during progressive hyperdynamic sepsis.

• Only subtle histological changes without signs of ATN occurred after 22 hours of peritonitis-induced septic shock.

• Renal venous congestion might be a hidden and clinically unrecognised factor contributing to the development of septic kidney dysfunction.

## Abbreviations

AKI: acute kidney injury; ATN: acute tubular necrosis; CO: cardiac output; CVP: central venous pressure; H&E: haematoxylin and eosin; IL: interleukin; ITBV: intrathoracic blood volume; MAP: mean arterial pressure; NOx: nitrate/nitrite; PAOP: pulmonary artery occlusion pressure; PCO2: partial pressure of carbon dioxide; PO2: partial pressure of oxygen; RVR: renal venous resistance; SVR: systemic vascular resistance; TBARS: thiobarbituric acid reactive species; TNF-α: tumour necrosis factor-α.

## Competing interests

The authors declare that they have no competing interests.

## Authors' contributions

RS and JC conducted the study, performed data collection, statistical analysis and helped to draft the manuscript. They contributed equally to this study. AK, JR, IN and TK helped to collect data and participated in the study design. VV conducted the study and performed data collection. OH performed histological analysis. PR contributed to the writing of the paper. MM conceived the study and contributed to the writing of the paper.

## Authors' information

Work was performed at the animal research laboratory of the 1^st ^Medical Department at Charles University Medical School.
